# TRIM56 restricts Coxsackievirus B infection by mediating the ubiquitination of viral RNA-dependent RNA polymerase 3D

**DOI:** 10.1371/journal.ppat.1012594

**Published:** 2024-09-30

**Authors:** Yao Wang, Yanyan Dong, Tian Luan, Yang Chen, Lexun Lin, Siwei Li, Danxiang Feng, Jianwei Wei, Yanru Fei, Guangtian Wang, Jiahui Pan, Yan Wang, Zhaohua Zhong, Wenran Zhao

**Affiliations:** 1 Department of Cell Biology, Harbin Medical University, Harbin, China; 2 Department of Microbiology, Harbin Medical University, Harbin, China; 3 Teaching Center of Pathogenic Biology, Harbin Medical University, Harbin, China; The Ohio State University, UNITED STATES OF AMERICA

## Abstract

Coxsackievirus B (CVB) is the major causative pathogen for severe diseases such as viral myocarditis, meningitis, and pancreatitis. There is no effective antiviral therapy currently available for CVB infection primarily due to that the pathogenesis of CVB has not been completely understood. Viruses are obligate intracellular pathogens which subvert cellular processes to ensure viral replication. Dysregulation of ubiquitination has been implicated in CVB infection. However, how ubiquitination is involved in CVB infection remains unclear. Here we found that the 3D protein of CVB3, the RNA-dependent RNA polymerase, was modified at K220 by K48-linked polyubiquitination which promoted its degradation through proteasome. Proteomic analysis showed that the E3 ligase TRIM56 was upregulated in CVB3-infected cells, while the majority of TRIMs remained unchanged. Pull-down and immunoprecipitation analyses showed that TRIM56 interacted with CVB3 3D. Immunofluorescence observation showed that viral 3D protein was colocalized with TRIM56. TRIM56 overexpression resulted in enhanced ubiquitination of CVB3 3D and decreased virus yield. Moreover, TRIM56 was cleaved by viral 3C protease in CVB3-infected cells. Taken together, this study demonstrated that TRIM56 mediates the ubiquitination and proteasomal degradation of the CVB3 3D protein. These findings demonstrate that TRIM56 is an intrinsic cellular restriction factor against CVB infection, and enhancing viral protein degradation could be a potential strategy to control CVB infection.

## Author summary

Coxsackievirus B (CVB) is the major causative agent for a range of human diseases. As small infectious agent with a simple genome, CVB relies on the subversion of host processes to replicate. On the other hand, host cells contain antiviral strategies to restrict CVB replication. The present study aims to address the role played by a cellular protein termed TRIM56 in the infection of CVB3, a representative serotype of CVB, which is the causative agent of inflammatory heart disease. Our study found that viral RNA replicase 3D harbors a type of covalent modification termed polyubiquitination, resulting in the accelerated degradation of this viral replicase. We show that TRIM56 is the cellular enzyme which promotes the polyubiquitination of viral 3D protein. We demonstrated that when TRIM56 was overexpressed in the cell, the production of viral particles was significantly reduced. Moreover, we found that the protease 3C of CVB3 counteracts the antiviral effect of TRIM56 through its protease activity. Together, these data demonstrate that TRIM56 is an intrinsic cellular restriction factor against CVB infection. Enhancing viral protein degradation could be a potential strategy to control CVB infection.

## 1. Introduction

Group B Coxsackieviruses (CVB) are the important causative pathogen for severe diseases such as viral myocarditis, meningitis, and pancreatitis [1–4]. Clinical study showed that the genomic RNA of CVB3 was detected in the blood and/or pericardial fluid of up to 30% of the hospitalized patients diagnosed as myocarditis or dilated cardiomyopathy [5]. CVB are of non-enveloped, positive-sensed single-stranded RNA viruses, belonging to *Enterovirus* genus of *Picornaviridae* family [2]. There are at least six serotypes of CVB recognized: CVB1, CVB2, CVB3, CVB4, CVB5, and CVB6. The genome of CVB contains a large open reading frame (ORF) flanked by a 5’-untranslated region (UTR) and 3’UTR with a poly(A) tail. The ORF encodes a single polyprotein which is autocleaved to produce four structural proteins (VP1 to VP4) and seven non-structural proteins (2A, 2B, 2C, 3A, 3B, 3C, and 3D). 3D is the RNA-dependent RNA polymerase (RdRp), which is responsible for the genomic replication of CVB [2,6,7].

Ubiquitination is one of the most prevalent forms of protein post-translational modifications, which is involved in almost all cellular processes [8]. Protein ubiquitination serves multiple functions, which are determined by the particular type of ubiquitin (Ub) linkage [9]. Ub is a small protein which contains seven lysines (K) in its total 76 amino acids [8]. The functional complexity of protein ubiquitination arises from that Ub can form distinct polymers via one of its seven K residues or the amino terminus [8]. It is well characterized that K48-linked polyubiquitination predominantly targets the ubiquitinated proteins to degradation in proteasomes, while K63-linked polyubiquitination alters the function or subcellular localization of the target proteins [10]. Ubiquitination is an enzymatic cascade involving the Ub-activating enzyme E1, Ub-conjugating enzyme E2, and Ub ligase E3 [8]. As the direct mediator for tagging Ub to substrates and for Ub chain elongation, E3 ligase is the primary component for substrate selection [11]. Because of the prevalence and importance of ubiquitination, some viruses are evolved to interfere with host ubiquitination-dependent signaling or to utilize this mechanism to evade innate immunity and to facilitate viral infection [12–14]. Previous studies have implicated that CVB3 replication utilized ubiquitination [15, 16], and targeting ubiquitin-proteasome system (UPS) suppressed viral replication [16, 17]. However, how ubiquitination is involved in CVB3 replication has not been fully understood.

Tripartite motif (TRIM) proteins form a large E3 ligase family consisting of more than 80 members in human genome [18–20]. TRIM proteins share three conserved domains consisting a RING finger, one or two B-box, and a coiled-coil (CC) domain on the N-terminal region of these proteins, which is collectively termed RBCC domain. The C-terminal regions of TRIMs are variable, which classify TRIMs into 11 subfamilies [18, 21–23]. The RBCC motif, which confers the E3 ligase activity of TRIMs, is the defining characteristic of TRIM family, while the C-terminal region plays a role in substrate recognition and binding [18, 21, 24, 25]. TRIMs are involved in various cellular activities such as apoptosis, autophagy, and innate immunity [18, 21, 26]. Some TRIM E3 ligases, including TRIM21, TRIM22, TRIM25, and TRIM52, show antiviral properties through directly targeting viral proteins to proteasomal degradation or re-establishing interferon (IFN) production [27–31]. These TRIMs are considered as the important constituents of cellular intrinsic antiviral effectors [18, 21]. TRIM56 shows antiviral effect against viruses from the families of *Flaviviridae*, *Coronaviridae*, and *Orthomyxoviridae* [32–34]. However, it is unknown whether or not TRIM56 exhibits antiviral activity against CVB3 infection.

In this study, we initially aimed to understand the precise role of UPS in CVB3 infection. We found that the 3D protein of CVB3 was modified at K220 by K48-linked ubiquitination and degraded through proteasomes. We further identified that the E3 ligase TRIM56 mediated the ubiquitination of 3D. Moreover, viral protease 3C cleaves TRIM56. Our study provides novel insights for understanding the interaction between CVB3 and the host cell.

## 2.Results

### 2.1. The RNA dependent RNA polymerase 3D of CVB3 is polyubiquitinated

Accumulating evidence has shown that ubiquitination is manipulated by a variety of viruses to facilitate the various steps of viral life cycle or to overcome the intracellular defense machinery [35]. The ubiquitination of viral replication enzymes or replication cofactors could facilitate viral genome replication [36, 37]. Conversely, ubiquitination is also used by the host to degrade viral proteins or to strengthen innate immunity [38, 39]. It has been implicated that the 3D protein of CVB3 is ubiquitinated during viral infection [16]. However, the detailed mechanism for 3D ubiquitination and the E3 ligase which mediates this process remain unknown.

**Fig 1:**
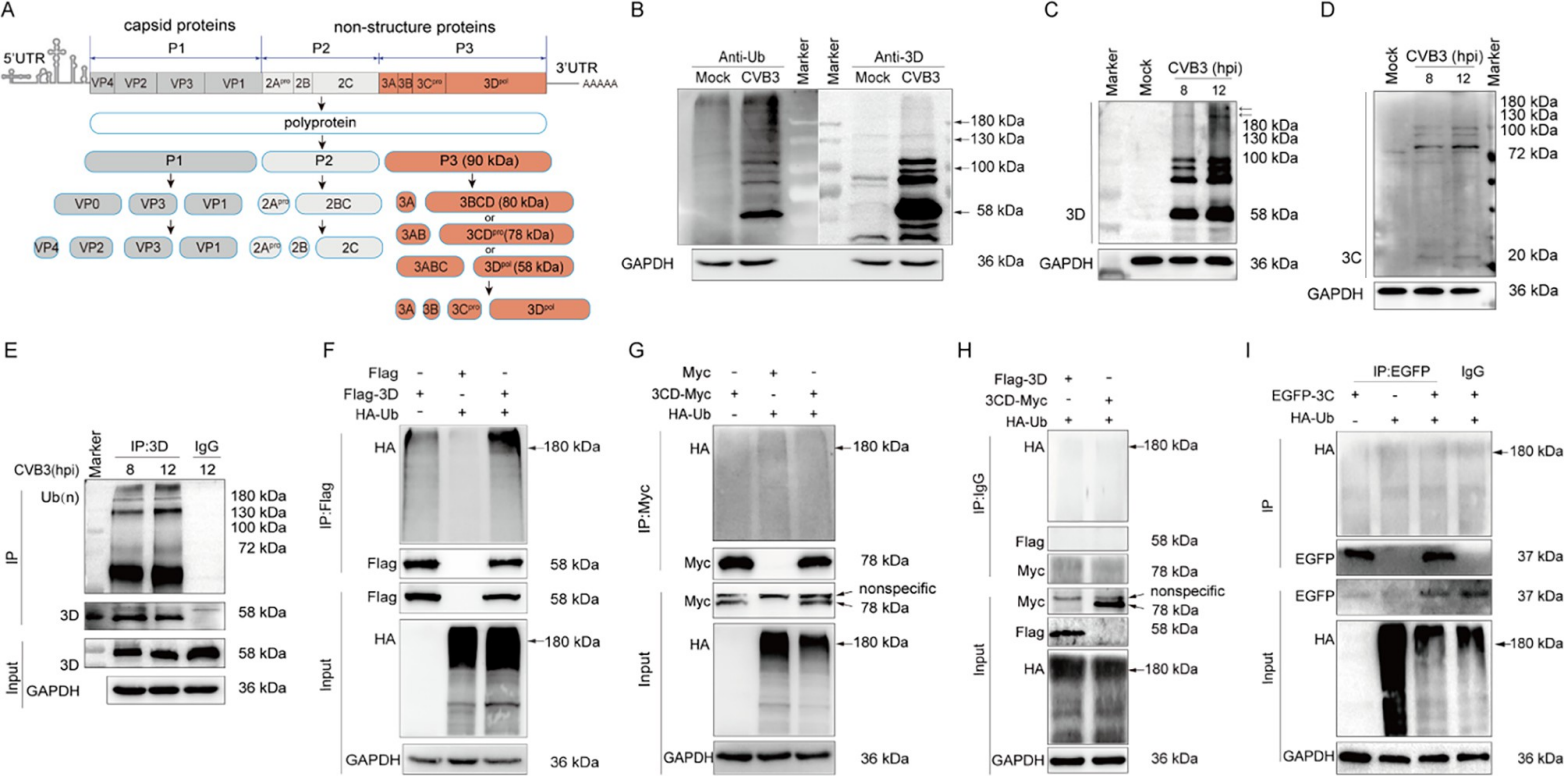
The RNA polymerase 3D of CVB3 is polyubiquitinated. (A) The schematic diagram of CVB3 genome organization and polyprotein processing. The processing intermediates of P3 were highlighted. The estimated MW of the processing intermediates containing both 3D and 3C (P3, 3BCD, and 3CD) was presented. (B) HeLa cells were infected with CVB3 at MOI of 1 for 12 h. Cells were collected and cell lysates were subjected to immunoblotting with anti-Ub and anti-3D antibodies to analyze the ubiquitination status of the cells and 3D protein. (C, D) HeLa cells were infected with CVB3 at MOI of 10 for 8 h or 12 h, and viral 3D and 3C were determined by immunoblotting with anti-3D (C) or anti-3C (D) antibody. (E) HeLa cells were infected with CVB3 at MOI of 10 for 12 h. Cell lysates were harvested at 8 and 12 h of p.i. and subjected to denatured co-IP analysis with anti-3D antibody. (F) HEK293T cells were co-transfected with pFlag-3D and pHA-Ub for 48 h. Total cellular proteins were extracted and subjected to co-IP with anti-Flag antibody under denatured condition. Ubiquitinated 3D was determined by immunoblotting with anti-HA antibody. (G) HEK293T cells were co-transfected with p3CD-Myc and pHA-Ub for 48 h. Total cellular proteins were extracted and subjected to denatured co-IP with anti-Myc antibody. Ubiquitinated 3D was determined by immunoblotting with anti-HA antibody. (H) HEK293T cells were co-transfected with pHA-Ub together with pFlag-3D or p3CD-Myc for 48 h. Total cellular proteins were extracted and subjected to denatured co-IP with normal IgG, followed by immunoblotting with anti-HA antibody. (I) HEK293T cells were co-transfected with pEGFP-3C and pHA-Ub for 48 h. Cells were collected and subjected to denatured IP with anti-EGFP antibody. Ubiquitinated 3C was determined by immunoblotting with anti-HA antibody. Experiments were repeated three times, and representative results were presented (B-I).

We started this study by validating the ubiquitination status of CVB3 3D protein. Since polyubiquitination is ubiquitous in eukaryotic cells [8], we made the hypothesis that 3D of CVB3 is modified by polyubiquitination. If that it is case, the most obvious change for ubiquitinated 3D would be the higher molecular weight (MW) than the 3D without Ub modification. However, it is worth to note that 3D protein with high MW presented by Western blotting is not necessarily the ubiquitinated 3D. During the translation of CVB, viral polyprotein is proteolytically processed into individual viral structural and non-structural proteins by viral proteases 2A^pro^, 3C^pro^, and 3CD^pro^ (Fig 1A) [40, 41]. The processing of enterovirus P3 not only generates individual mature viral proteins, but also generates cleavage intermediates: 3AB, 3CD, 3BCD, and 3ABC [42, 43]. Therefore, the viral intermediate proteins which harbor 3D may include 3CD, 3BCD, and 3ABCD (P3). According to the predicted MW, any of these precursors of P3 would be less than 100 kDa (Fig 1A) (GenBank: U57056.1). Theoretically, the entire viral polyprotein with an estimated MW of 244 kDa (containing 2185 aa residues) might also be detected by 3D antibody.

To determine the linkage of Ub chain with the 3D protein of CVB3, HeLa cells were infected with CVB3 at MOI of 1 for 12 h. The cell lysates were subjected to immunoblotting to analyze the overall ubiquitination status of the cells and viral 3D protein (Fig 1B). We found that the overall level of the ubiquitinated proteins was obviously increased in CVB3-infected cells (Fig 1B, upper left blot). There were proteins migrating at MW higher than 58 kDa (the MW of 3D monomer) that were identified by anti-3D antibody (Fig 1B, upper right blot), suggesting that these proteins are either the modified 3D proteins of CVB3 or viral precursor proteins which contain 3D.

To further define the 3D-containing proteins with MW higher than 58 kDa, HeLa cells were infected with CVB3, and viral proteins were determined by anti-3D (Fig 1C) and anti-3C antibodies (Fig 1D). In CVB3-infected cells, except the 3D monomer (58 kDa), there were proteins migrating between 72 and 100 kDa (Fig 1C). Moreover, high MW proteins (>180 kDa) were also detected by anti-3D antibody (Fig 1C; indicated by arrows). In contrast, proteins which were identified by anti-3C antibody showed MW from >72 kDa to about 100 kDa (Fig 1D). The 3C monomer was shown at 20 kDa (Fig 1D). We also noted the similar banding patterns between the blots probed by anti-3D and anti-3C antibodies at >70~100 kDa (Fig 1C and 1D), indicating that these proteins contain both 3D and 3C, which are likely the precursor proteins including 3CD, 3BCD, and 3ABCD. These data also indicate that proteins with MW at above 180 kDa detected by 3D antibody (Fig 1C) did not contain 3C of CVB3.

Denatured co-IP analysis identified that 3D proteins migrating at high MW (>72 kDa) were covalently linked to polyubiquitin chains in CVB3-infected cells (Fig 1E). The ubiquitination of 3D protein of CVB3 was further validated in the cells co-transfected with Flag-tagged 3D and HA-tagged Ub, followed by co-IP analysis (Fig 1F). We show that 3D of CVB3 was present primarily as high MW molecules (>180 kDa) (Fig 1F), indicating the polyubiquitination status of 3D. In contrast, the ubiquitination of 3CD was absent (Fig 1G). The specificity of the antibodies used in co-IP was also determined (Fig 1H), further indicating the reliability of these results. Finally, we show that EGFP-tagged 3C was not ubiquitinated (Fig 1I), demonstrating that ubiquitination exclusively occurs in the 3D protein of CVB3. These observations also implicate that it is unlikely that the polyubiquitination process occurs at the 3CD-containing viral precursors (3ABCD and 3BCD), since 3CD was not ubiquitinated. Collectively, these results show that viral 3D protein is polyubiquitinated in CVB3-infected cells.

### 2.2. The 3D protein of CVB3 is modified by K48-linked polyubiquitin chain

**Fig 2:**
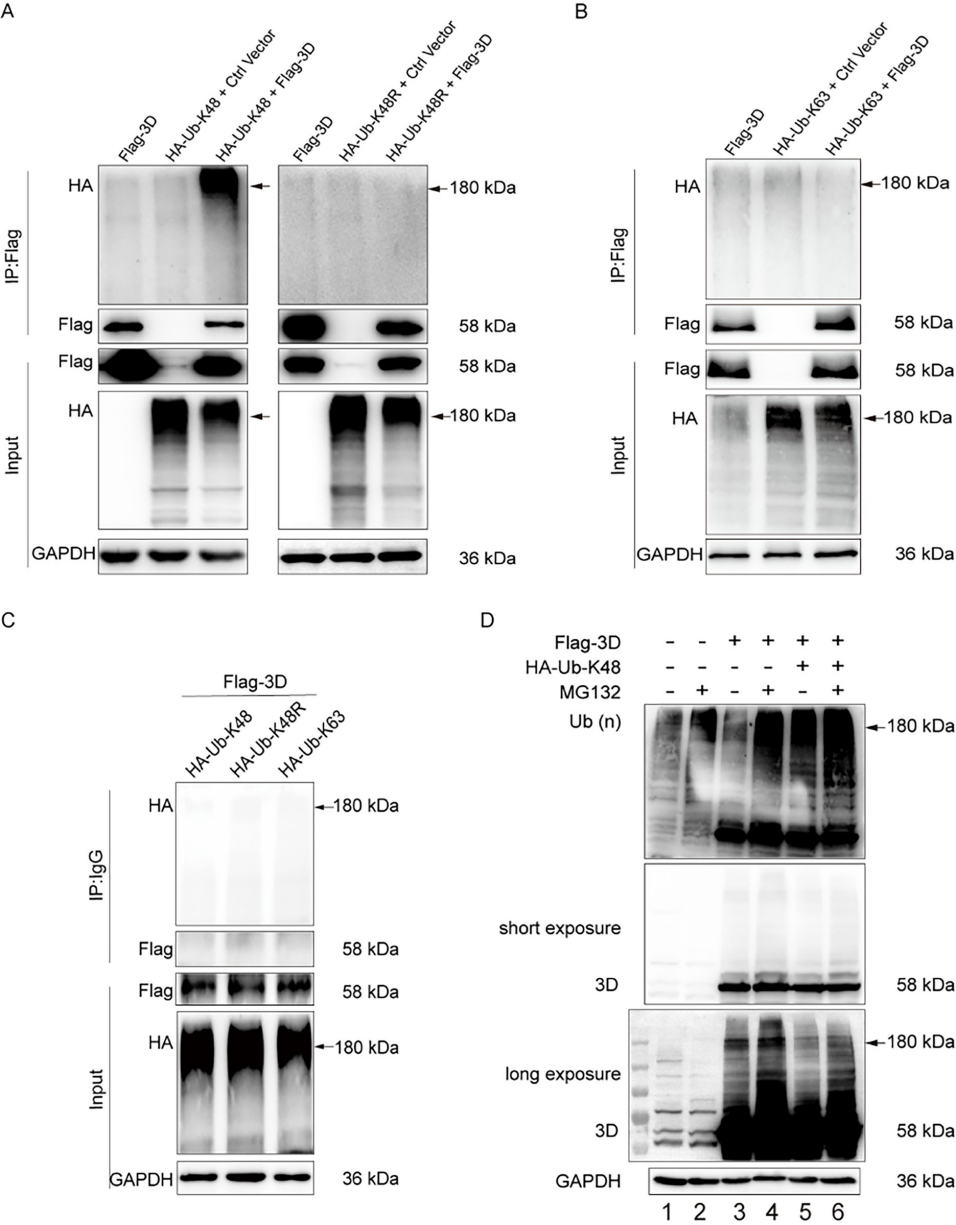
The 3D protein of CVB3 is modified by K48-linked polyubiquitin chain. (A) HEK293T cells were co-transfected with pFlag-3D and pHA-Ub-K48 or pHA-Ub-K48R for 48 h. Total cellular proteins were extracted and subjected to denatured IP with anti-Flag antibody. Ubiquitinated 3D was determined by immunoblotting with anti-HA antibody. (B) HEK293T cells were co-transfected with pFlag-3D and pHA-Ub-K63 for 48 h. Cells were collected and cell lysates were subjected to denatured IP with anti-Flag antibody. Ubiquitinated 3D was determined by immunoblotting with anti-HA antibody. (C) HEK293T cells were co-transfected with pFlag-3D together with pHA-Ub-K48 or pHA-Ub-K48R or pHA-Ub-K63 for 48 h. Cells were collected and cell lysates were subjected to denatured IP with normal IgG, followed by immunoblotting with anti-HA antibody. Ubiquitinated 3D was determined by immunoblotting with anti-HA antibody. (D) HeLa cells were co-transfected with pFlag-3D and pHA-Ub-K48 for 24 h, followed by the treatment of MG132 for 6 h. Cell lysates were extracted and subjected to Western blotting with anti-Flag antibody. Experiments were repeated three times, and representative results were presented (A-D). Ctrl Vector: The empty vector which expresses Flag tag.

To identify the type of Ub chain linked to the 3D protein of CVB3, we first examined the K48-linked ubiquitination, given that K48 linkage of ubiquitination is predominant in the cell which controls protein degradation [44, 45]. To this end, HEK293T cells were co-transfected with pFlag-3D and the plasmid expressing HA-tagged Ub-K48 (pHA-Ub-K48) for 48 h (Fig 2A, left blots). In the HA-Ub-K48, the lysine residue of K48 was maintained while other lysine residues of Ub were mutated. A plasmid expressing HA-Ub-K48R (pHA-Ub-K48R), in which K48 was mutated to arginine (R) while the remaining lysine residues were unchanged, was used as control (Fig 2A, right blots). Cell lysates were extracted and subjected to denatured IP with anti-Flag antibody, followed by immunoblotting with anti-HA antibody (Fig 2A). The immunoprecipitated proteins (Flag-3D) with MW around 180 kDa were identified by anti-HA antibody in the cells co-expressing HA-Ub-K48 and Flag-3D (Fig 2A: left top blot; indicated by arrow). In contrast, no immunoprecipitated proteins (Flag-3D) were identified by anti-HA antibody in the cells co-expressing HA-Ub-K48R and Flag-3D (Fig 2A: right top blot; indicated by arrow). These data support that the 3D of CVB3 is modified by K48-linked Ub chain. We also excluded K63-linkage of the Ub chain in the 3D of CVB3 by denatured IP in the cells co-expressing Flag-3D and HA-Ub-K63 (Fig 2B), in which all lysine residues of Ub were mutated except K63. To ensure the specificity of the co-IP results, normal IgG was utilized at the same time when anti-Flag antibody was applied in denatured IP (Fig 2C). We show that there is no non-specific interaction between antibody and Flag-3D (Fig 2C).

Since the role of K48-linked ubiquitination is primarily to target proteins to proteasomal degradation [44, 45], we determined the outcome of the K48-linked ubiquitination of the 3D of CVB3. To this end, HeLa cells were co-transfected with pFlag-3D and pHA-Ub-K48 for 24 h, followed by the treatment of MG132, the proteasome inhibitor, for 6 h. With MG132 treatment, 3D proteins migrating at and above 180 kDa were obviously accumulated (Fig 2D; blot at bottom: lane 4 vs lane 3), suggesting that 3D is polyubiquitinated and degraded through proteasome. In the cells overexpressing HA-Ub-K48 (Fig 2D; blot at bottom: lane 5), the amount of 3D proteins around 180 kDa was dramatically reduced compared to that in the cells without Ub-K48 overexpression (Fig 2D; blot at bottom: lane 3). With the treatment of MG132, the amount of the 3D proteins with WM around 180 kDa was increased (Fig 2D; blot at bottom: lane 6 vs lane 5). Collectively, these observations indicate that the 3D of CVB3 is subjected to K48-linked polyubiquitination and proteasomal degradation.

### 2.3. The 3D of CVB3 is polyubiquitinated at K220

**Fig 3:**
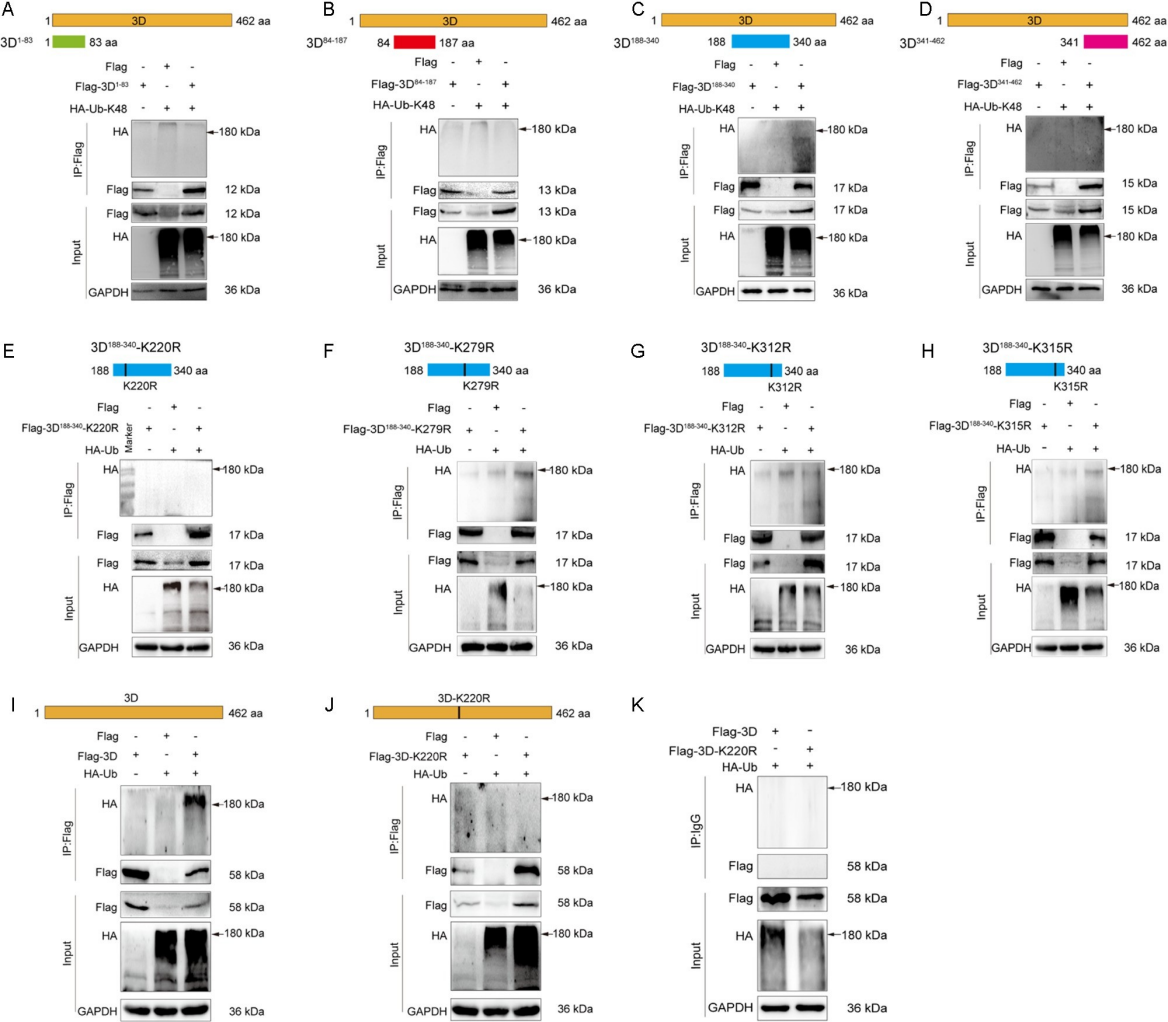
CVB3 3D is polyubiquitinated at K220. (A-D) The constructs expressing truncated 3D of CVB3 (pFlag-3D^1-83^, pFlag-3D^84-187^, pFlag-3D^188-340^, and pFlag-3D^341-462^) were generated. HEK293T cells were co-transfected with pHA-Ub-K48 and the plasmid expressing each of the truncated 3D for 48 h. Total cellular proteins were extracted and subjected to denatured IP with anti-Flag antibody. The ubiquitination of the truncated 3D was determined by immunoblotting with anti-HA antibody. (E-H) Each of the four lysine residues (K220, K279, K312, and K315) in Flag-3D^188-340^ was mutated individually. HEK293T cells were co-transfected with pHA-Ub and each of the plasmids expressing the mutated Flag-3D^188-340^ for 48 h. Cell lysates were analyzed by denatured IP with anti-Flag antibody. The ubiquitination of the 3D^188-340^ mutants were determined by immunoblotting with anti-HA antibody. (I, J) HEK293T cells were co-transfected with pHA-Ub and pFlag-3D (I) or pFlag-3D-K220R (J) for 48 h. Cell lysates were subjected to denatured IP with anti-Flag antibody followed by immunoblotting with anti-HA antibody. (K) HEK293T cells were co-transfected with pHA-Ub and pFlag-3D or pFlag-3D-K220R for 48 h. Cell lysates were subjected to denatured IP with normal IgG followed by immunoblotting with anti-HA antibody. All of the experiments were repeated three times, and representative results were presented.

To determine the amino acid residues in the 3D of CVB3 which are linked to Ub chain, we generated four constructs expressing truncated 3D containing the amino acid residues of 1–83, 84–187, 188–340, and 341–462. Co-expression of each of the truncated 3D construct with HA-Ub-K48 was performed in HEK293T cells for 48 h, followed by denatured IP to determine the Ub linkage site (Fig 3A–3D). Ubiquitinated proteins with MW of less than 180 kDa were identified in the cells expressing Flag-3D^188-340^ (Fig 3C). The estimated MW of 3D^188-340^ without modification is 17 kDa, while the intact 3D is 58 kDa. Therefore, the ubiquitinated 3D^188-340^ showed MW less than 180 kDa. These findings suggest that 3D^188-340^ contains the amino acid residues which are linked to Ub chain.

To identify which of the four lysine residues (K220, K279, K312, K315) in the 3D^188-340^ truncate is responsible for linking Ub chain, we generated four 3D^188-340^ mutants, each of which contains a mutated lysine (K to R mutation) (Fig 3E–3H). HEK293T cells were co-transfected with the plasmids expressing mutated 3D^188-340^ and HA-Ub for 48 h (Fig 2E–2H). Denatured IP analysis showed that ubiquitinated 3D^188-340^ was absent in the cells expressing 3D^188-340^-K220R (Fig 3E), demonstrating that K220 is the lysine residue which links to polyubiquitin chain.

To validate the ubiquitination site of 3D, a plasmid expressing the full length of 3D with K220R mutation (pFlag-3D-K220R) was generated based on the plasmid expressing the wild-type 3D (pFlag-3D) (Fig 3J). HEK293T cells were co-transfected with pHA-Ub and pFlag-3D or pFlag-3D-K220R for 48 h. Co-IP showed that wild type 3D was ubiquitinated (Fig 3I), while 3D-K220R was not (Fig 3J). We show that there was no non-specific interaction between antibody and Flag-3D (Fig 3K). Collectively, these results demonstrate that the 3D of CVB3 is polyubiquitinated at K220.

### 2.4. E3 ligase TRIM56 is up-regulated in response to CVB3 infection

**Fig 4:**
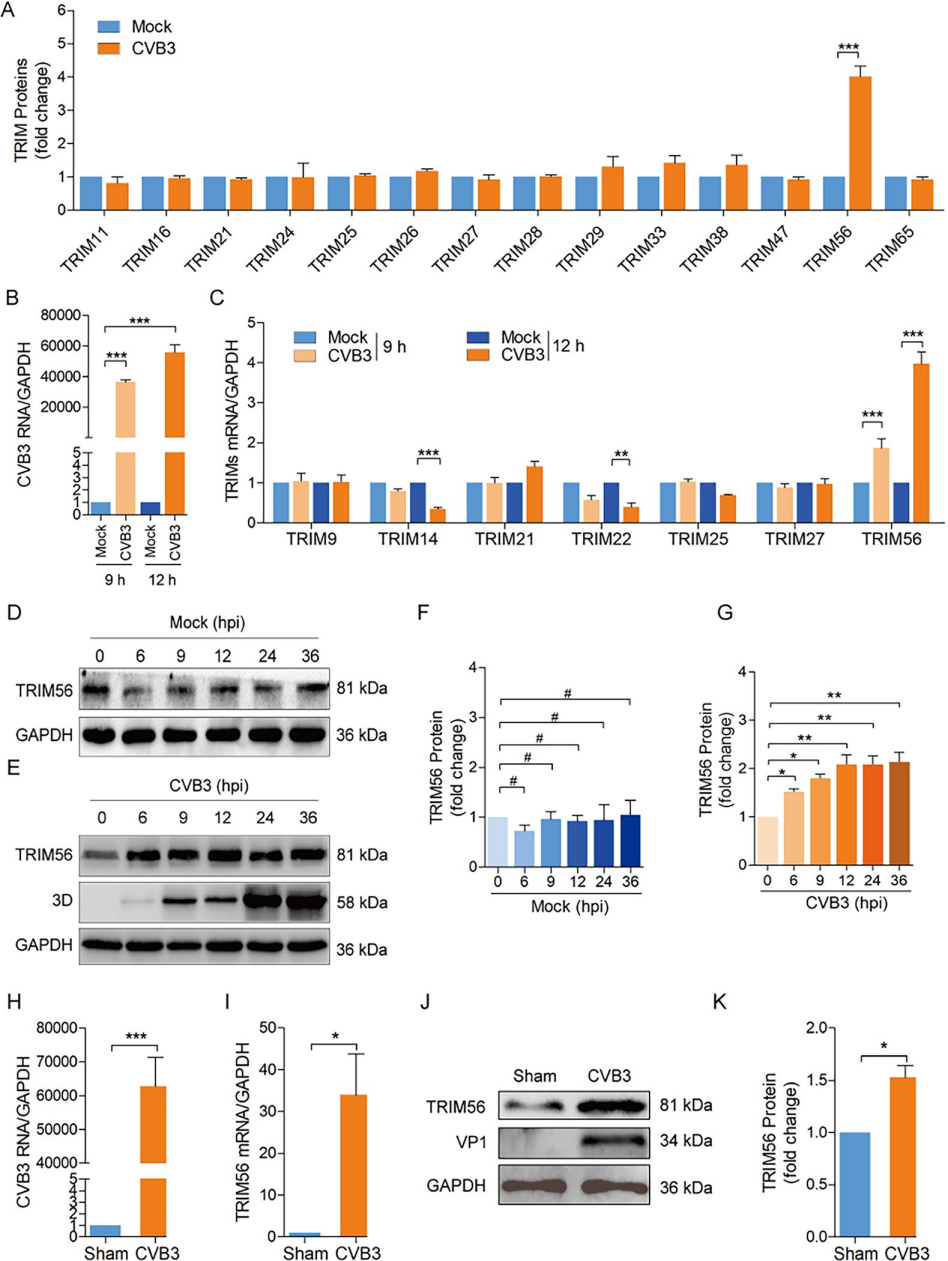
E3 ligase TRIM56 is up-regulated in response to CVB3 infection. (A) HeLa cells were mock-infected or infected with CVB3 at MOI of 1 for 24 h. Total cellular proteins were extracted and analyzed by mass spectrometry. The protein levels of TRIMs from the results of mass spectrometry (S1 Table) were analyzed. (B, C) HeLa cells were infected with CVB3 at MOI of 1 for 12 h. Total RNA was extracted and analyzed by RT-qPCR to determine the mRNA abundance of the genomic RNA level of CVB3. (B) and the selected members of the TRIM family (C). (D-G) HeLa cells were infected with CVB3 at MOI of 1 for 36 h. Cells were collected at various timepoints of p.i. and analyzed by immunoblotting. Experiments were repeated three times, and representative results were presented (B-G). (H-K) Newborn Balb/c mice were infected with 10^6^ TCID_50_ of CVB3. RNA and proteins were extracted from the myocardium of the mice at day 5 of p.i. and analyzed by RT-qPCR (H and I) or immunoblotting (J and K). *n* = 6. *, *P* < 0.05; ***, *P* < 0.001. #: no significant. Data are presented as mean ± SEM.

With the identification of the ubiquitination of 3D of CVB3, we set out to identify the E3 ligase which is responsible for 3D ubiquitination. To this end, we carried out proteomics study to show the protein expression profile in the cells infected with CVB3. HeLa cells were infected with CVB3 at MOI of 1 for 24 h, and the total proteins were extracted and analyzed by mass spectrometry (MS). We found that TRIM56 E3 ligase was significantly increased in the cells infected with CVB3, while the majority of TRIMs remain unchanged (Fig 4A and S1 Table). To validate the data of MS, the expression of TRIM56 and other TRIMs, which have been implicated in viral infection [46, 47], was determined for CVB3-infected cells with RT-qPCR at various timepoints of post-infection (p.i.) (Fig 4B and 4C). We found that the mRNA abundance of TRIM56 increased in time-dependent manner, while other TRIMs were either unchanged or declined (Fig 4B and 4C). The protein levels of TRIM56 were also increased in the cells infected with CVB3 (Fig 4D–4G). To further validate the correlation between TRIM56 and CVB3 infection, newborn Balb/c mice were infected with CVB3 (1.1 × 10^7^ TCID_50_), and the expression of TRIM56 in mouse myocardium was determined at day 5 of p.i.. The results show that mRNA and protein levels of TRIM56 were significantly increased in the myocardium infected with CVB3 (Fig 4H–4K). Collectively, these *in vitro* and *in vivo* data support that TRIM56 expression was increased in response to CVB3 infection.

### 2.5. TRIM56 interacts with the 3D of CVB3

**Fig 5:**
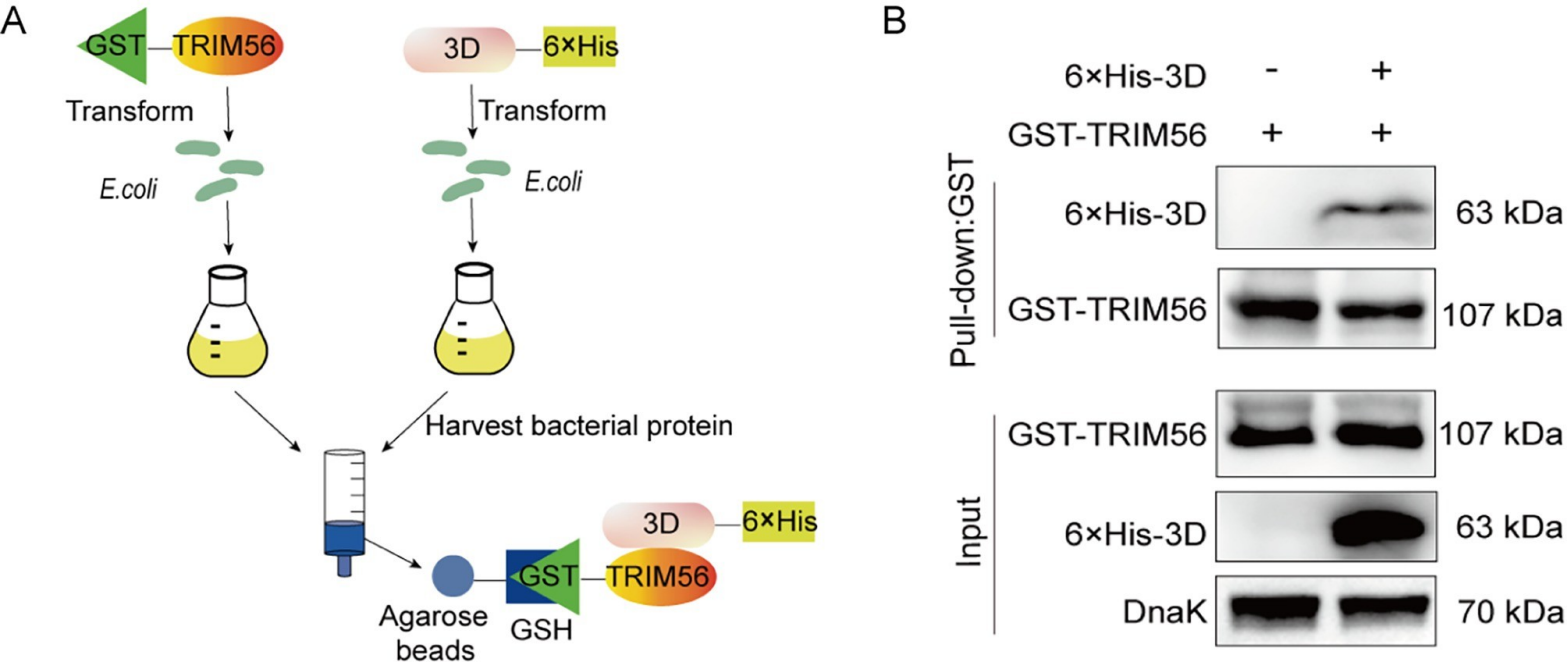
TRIM56 interacts with the 3D of CVB3. (A) The schematic diagram shows how pulldown assay was performed. GST-TRIM56 was expressed *E*. *coli*, and bacterial proteins were extracted. *E*. *coli* (BL21) was transformed with p3D-6×His or control vector (pcDNA3.1–6×His) to express 6×His-tagged 3D. Bacterial proteins were harvested and incubated with GSH-agarose beats. The eluted proteins were analyzed by immunoblotting. (B) Immunoblots were performed to analyze the eluted proteins (A) with anti-His and anti-GST antibodies. Experiments were repeated three times, and representative results were presented.

Given that TRIM56 is a typical E3 ligase and the only TRIM protein identified with increased expression in response to CVB3 infection, we hypothesized that TRIM56 mediates 3D ubiquitination and degradation. To show the role of TRIM56 in the ubiquitination of 3D of CVB3, we first determined the interaction between TRIM56 and viral 3D protein. To this end, GST-tagged TRIM56 (GST-TRIM56) and 6×His-tagged 3D (3D-6×His) were expressed in *E*. *coli*, respectively (Fig 5A). Bacterial proteins were harvested and subjected to pull-down assay (Fig 5A and 5B). We show that the 3D protein of CVB3 interacts with TRIM56 (Fig 5B).

### 2.6. TRIM56 colocalized with the 3D protein of CVB3

**Fig 6:**
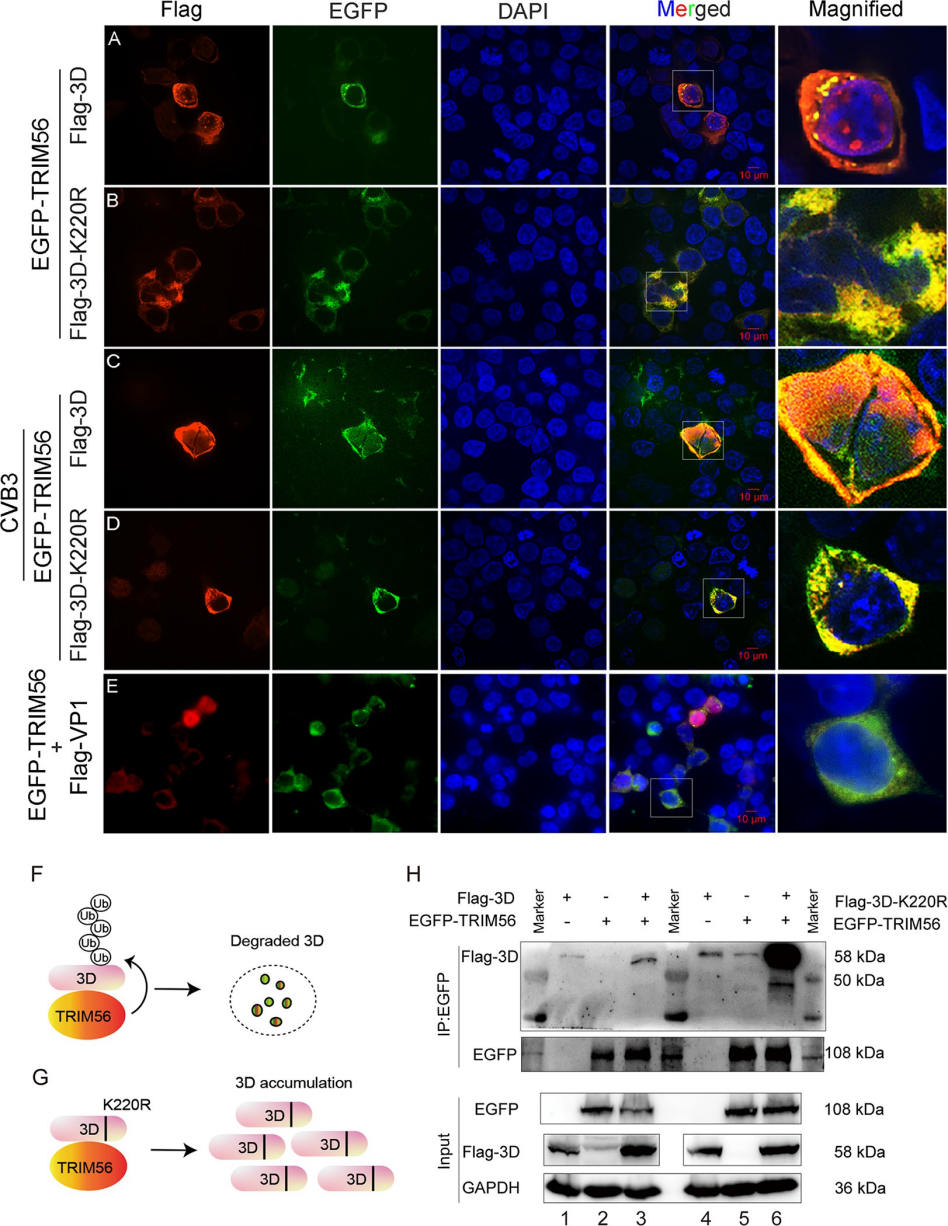
TRIM56 colocalizes with the 3D protein of CVB3. (A, B) HEK293T cells were co-transfected with pEGFP-TRIM56 together with pFlag-3D (A) or pFlag-3D-K220R (B) for 24 h. Cells were vitalized with anti-Flag in confocal microscopy. (C, D) HEK293T cells were co-transfected with pEGFP-TRIM56 together with pFlag-3D (C) or pFlag-3D-K220R (D) for 24 h, followed the infection of CVB3 (MOI = 1) for 12 h. Cells were observed in confocal microscopy. (E) HEK293T cells were co-transfected with pFlag-VP1 and pEGFP-TRIM56 for 24 h. Cells were visualized in confocal microscopy with anti-Flag antibody. (F, G) The schematic diagrams show that TIRM56 mediates the ubiquitination and degradation of CVB3 3D (A, C). Mutated 3D (3D-K220R), which is not ubiquitinated, accumulates in the cells (B, D). (H) HEK293T were co-transfected with EGFP-TRIM56 and Flag-3D (or Flag-3D-K220R) for 24 h. Cell lysates were harvested and subjected to co-IP analysis.

To further demonstrate that TRIM56 interacts with the 3D protein of CVB3, HEK293T cells were co-transfected with pEGFP-TRIM56 and pFlag-3D (or pFlag-3D-K220R) for 24 h. The co-localization of TRIM56 and 3D was observed with fluorescence microscopy (Fig 6A and 6B). We show that TRIM56 colocalized with 3D protein in the cytoplasm (Fig 6A), while the co-localization of TRIM56 with 3D-K220R was significantly enhanced (Fig 6B), compared with the cells expressing wild type 3D (Fig 6A). The co-localization of TRIM56 and viral protein 3D was also determined in CVB3-infected cells (Fig 6C and 6D). HEK293T cells were transfected with pFlag-3D or pFlag-3D-K220R for 24 h, followed by the infection of CVB3 at MOI of 1 for 8 h. The results show that TRIM56 was co-localized with either wild type or mutated 3D in the context of CVB3 infection (Fig 6C and 6D). The co-localization between TRIM56 and 3D-K220R (Fig 6B and 6D) was obviously enhanced compare with that between TRIM56 and wild type 3D (Fig 6A and 6C). To show the specific interaction between TRIM56 and CVB3 3D, cells were co-transfected with pEGFP-TRIM56 and pFlag-VP1 for 24 h (Fig 6E). Cells were observed in fluorescence microscopy. We show that there is no interaction between TRIM56 and CVB3 VP1 (Fig 6E). To be observed that, as demonstrated previously [48], VP1 of CVB3 could also be translocated into the nucleus (Fig 6E).

The interaction between TRIM56 and the 3D of CVB3 (or 3D-K220R) was also determined by co-IP (Fig 6F–6H). HEK293T cells were co-transfected with pFlag-3D (or Flag-3D-K220R) and pEGFP-TRIM56 for 48 h. IP was used to determine the interaction between TRIM56 and 3D. We show that both of the wild type 3D (Fig 6H, lane 3 on top blot) and 3D-K220R (Fig 6H, lane 6 on top blot) interact with TRIM56, but 3D-K220R was found to interact with TRIM56 in dramatically increased quantity (Fig 6H, lane 6 on top blot). These observations indicate that wild type 3D protein is ubiquitinated and degraded (Fig 6F), while 3D-K220R remains at high level due to the lack of ubiquitination and the reduced degradation (Fig 6G). Taken together, these data demonstrate that TRIM56 interacts with 3D of CVB3 and promotes 3D degradation.

### 2.7. TRIM56 promotes the ubiquitination and proteasomal degradation of CVB3 3D

**Fig 7:**
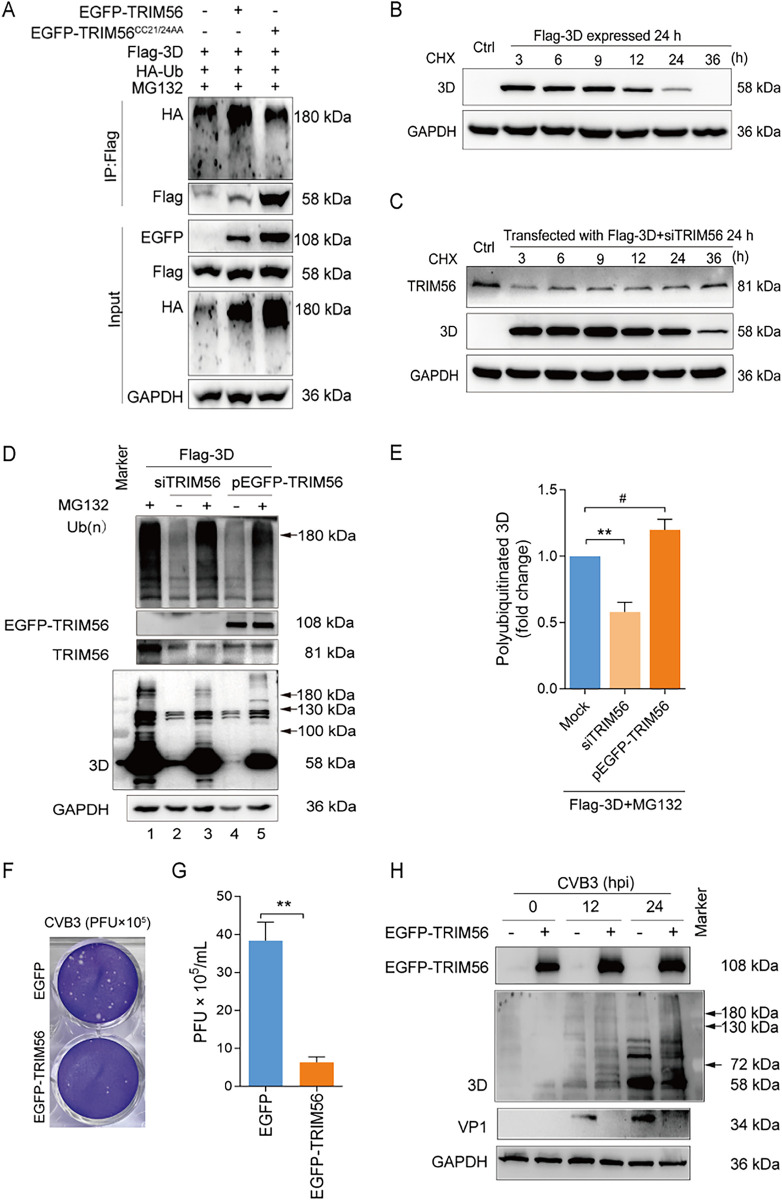
TRIM56 promotes the ubiquitination and proteasomal degradation of CVB3 3D. (A) HEK293T cells were co-transfected with the pFlag-3D, pHA-Ub, and pEGFP-TRIM56 or pEGFP-TRIM56^CC21/24AA^ for 24 h, followed by the treatment of MG132 for 6 h. Cell lysates were subjected to denatured IP with anti-Flag antibody and immunoblotting. (B) HEK293T cells were transfected with pFlag-3D for 24 h, followed by the treatment of CHX at 100 mg/mL. Cells were collected at various timepoints after CHX treatment and analyzed by immunoblotting. (C) HEK293T cells were co-transfected with pFlag-3D and siTRIM56 for 24 h, followed by the treatment of CHX at 100 mg/mL. Cells were collected at various timepoints after CHX treatment and analyzed by immunoblotting. (D, E) HeLa cells were co-transfected with pFlag-3D together with siTRIM56 or pEGFP-TRIM56 for 24 h, followed by the treatment with or without MG132 (20 μM) for 6 h. Cells were collected and subjected to immunoblotting. The overall ubiquitination status of the cells is shown with anti-Ub antibody (top blot). 3D was determined with anti-Flag antibody. (F, G) HeLa cells were transfected with pEGFP-TRIM56 for 24 h and then infected with CVB3 (MOI = 1). The cells were collected at 24 h of p.i. and subjected to three freeze-thaw circles to harvest viruses. Virions were determined by plaque assay using HeLa cells with 24 h of virus infection. (H) HeLa cells were transfected with pEGFP-TRIM56 for 24 h, followed by the infection of CVB3 (MOI = 1) for 24 h. Cells were collected at 12 h and 24 h of p.i. and analyzed by immunoblotting. Experiments were repeated three times, and representative results were presented. Data were presented as mean ± SEM. *, *P* < 0.05; **, *P* < 0.01; ***, *P* < 0.001. #: no significant. CHX: cycloheximide. hpi: hours of post-infection.

Our available data show that 3D protein was polyubiquitinated in CVB3-infected cells, and TRIM56 interacts with 3D and promotes 3D degradation. To confirm the role of TRIM56 in 3D ubiquitination, we further investigated how TRIM56 regulates the stability of 3D protein of CVB3. To this end, HEK293T cells were co-transfected with the construct expressing Flag-3D, HA-Ub, and EGFP-TRIM56 or mutated TRIM56 (EGFP-TRIM56^CC21/24AA^), which lacks the E3 ligase activity (Fig 7A). Cells were treated with MG132 to block the degradation of the ubiquitinated proteins through proteasomes. IP was carried out under denatured condition with anti-Flag to determine the 3D protein which is covalently linked to Ub chain. As shown in Fig 7A, in the cells co-expressing Flag-3D and HA-Ub, Flag-3D was existing primarily as ubiquitinated molecules migrating at >180 kDa (Fig 7A: top blot), as demonstrated above (Fig 3I). In the cells co-expressing Flag-3D, HA-Ub, and EGFP-TRIM56, the ubiquitination of Flag-3D was obviously enhanced (Fig 7A: top blot), compared with that in the cells without EGFP-TRIM56 overexpression, demonstrating that TRIM56 promotes 3D ubiquitination. Finally, in the cells co-expressing Flag-3D, HA-Ub, and EGFP-TRIM56^CC21/24AA^, there was still ubiquitinated Flag-3D which is similar in quantity to that in the cells with no transient expression of TRIM56. These data demonstrated that TRIM56 enhances the polyubiquitination of the 3D protein of CVB3.

To verify that TRIM56 promotes the polyubiquitination and degradation of the 3D protein of CVB3, the stability of 3D was determined by cycloheximide (CHX)-chase assay in the cells transfected with pFlag-3D (Fig 7B) or co-transfected with pFlag-3D and siTRIM56 (Fig 7C) for 24 h, followed by the treatment of protein synthesis inhibitor CHX. The protein level of 3D was determined at various time points after CHX treatment. We found that 3D abundance was significantly reduced at 24 h after the treatment of CHX in the cells without TRIM56 knockdown (Fig 7B). In contrast, in the cells with TRIM56 knockdown, 3D remained at high level at 24 h after CHX treatment (Fig 7C). When TRIM56 expression was recovered at 36 h after CHX treatment, 3D level was decreased (Fig 7C). Collectively, these results demonstrate that TRIM56 promotes the degradation of 3D of CVB3.

The role of TRIM56 in CVB3 3D polyubiquitination was further investigated in the cells with TIRM56 knockdown or TRIM56 overexpression. To this end, HeLa cells were co-transfected with either pFlag-3D and siTRIM56 or pFlag-3D and pEGFP-TRIM56 for 48 h, followed by the treatment of proteasome inhibitor MG132 for 6 h to block the degradation of the ubiquitinated 3D (Fig 7D and 7E). The total ubiquitinated proteins (Fig 7D: top blot) and 3D were examined by immunoblotting. We show that 3D protein migrating at >100 kDa was dramatically reduced in the cells with TRIM56 knockdown (Fig 7D, lane 3 vs lane 1; Fig 7E), indicating that TRIM56 promotes 3D polyubiquitination and proteasomal degradation. Moreover, 3D protein migrating at high MW (>180 kDa) appeared in the cells overexpressing TRIM56 (Fig 7D: lane 5; Fig 7E), supporting that TRIM56 promotes 3D polyubiquitination.

To further reveal the role played by TRIM56 in CVB3-infected cells, viral replication was determined in the cells with TRIM56 overexpression (Fig 7F–7H). We show that TRIM56 overexpression resulted in reduced virus yield (Fig 7F and 7G) and decreased levels of 3D and VP1 at 24 h of post-infection (Fig 7H). These data show that TRIM56 overexpression inhibits CVB3 replication. Taken together, these data demonstrate that TRIM56 mediates the polyubiquitination and proteasomal degradation of the 3D of CVB3 and suppresses viral replication.

### 2.8. TRIM56 is cleaved by the 3C protease of CVB3

**Fig 8:**
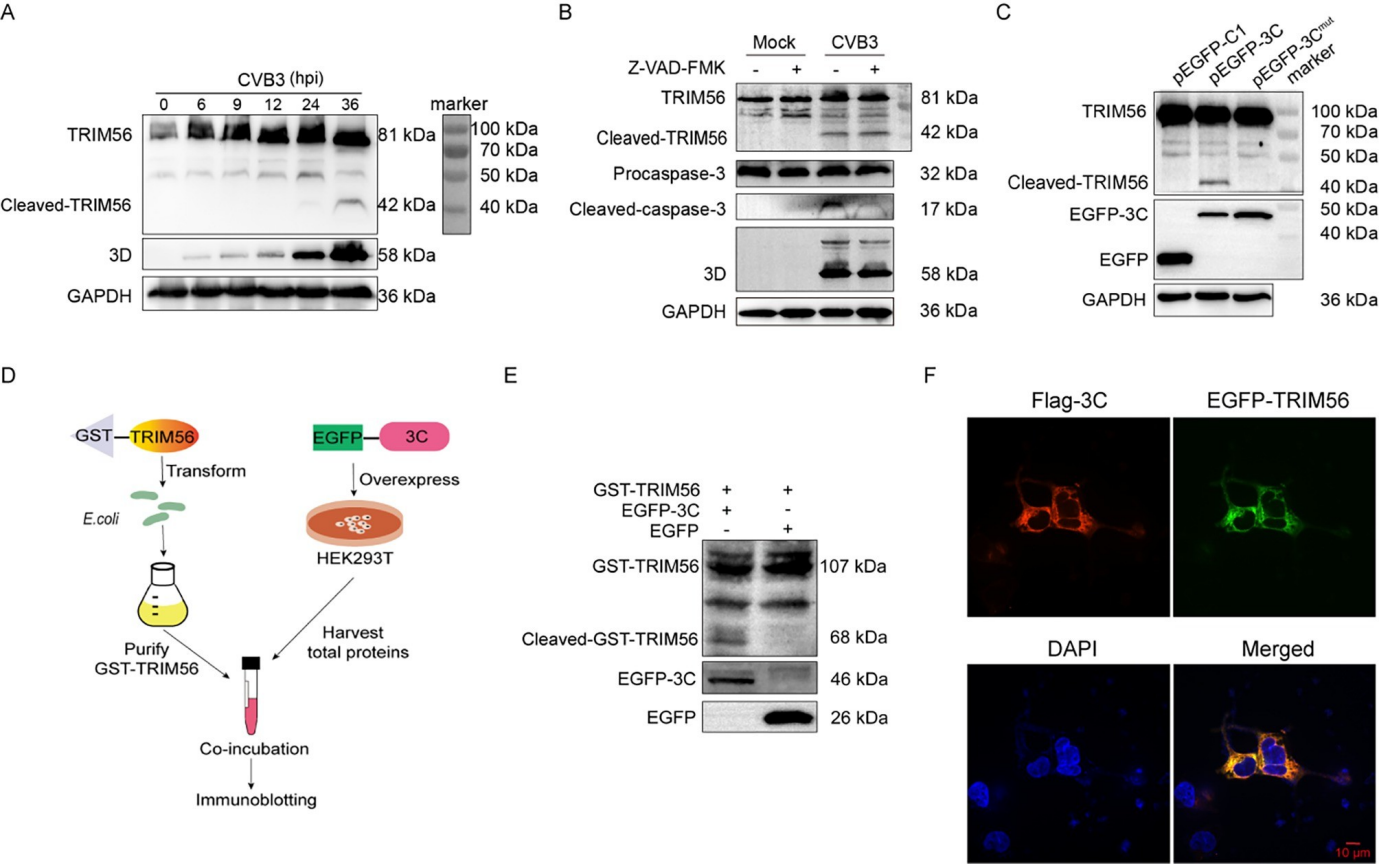
TRIM56 is cleaved by the 3C protease of CVB3. (A) HeLa cells were infected with CVB3 (MOI = 1). Cells were collected at various timepoints of p.i.. Cell lysates were analyzed by immunoblotting. (B) HeLa cells were mock-infected or infected with CVB3 for 24 h in the presence or absence of 20 μM Z-VAD-FMK, a pan-caspase inhibitor. Cell lysates were analyzed by immunoblotting. (C) HeLa cells were transfected with plasmids expressing EGFP-3C or EGFP-3C^mut^ (3C-C147A) for 24 h. Cell lysates were analyzed by immunoblotting. (D and E) Schematic diagram shows the interaction of TRIM56 with 3C protease of CVB3 *in vitro* (D). Bacterial proteins, which were extracted from *E*. *coli* transformed with GST-TRIM56, were incubated with the proteins extracted from the cells transfected with pEGFP-3C or pEGFP-C1 (control vector). The incubation mixture was analyzed by immunoblotting (E). (F) The colocalization of CVB3 3C and TRIM56 was observed by fluorescence microscopy. HeLa cells were co-transfected with pFlag-3C and pEGFP-TRIM56 for 24 h. Cells were fixed and observed by confocal microscopy. Nuclei were stained with DAPI. Experiments were repeated three times, and representative results were presented. hpi: hours of post-infection. hpi: hours of post-infection.

Thus far, we proposed that TRIM56 functions as the restriction factor to CVB3 infection through mediating the proteasomal degradation of the viral 3D protein. Therefore, we wondered how CVB3 could overcome the restriction of TRIM56, which was upregulated during CVB3 infection (Fig 4). It is established that the proteases of picornaviruses degrade cellular proteins to counteract host antiviral defense [49]. We hypothesized that TRIM56 may also be cleaved by the proteases of CVB3. In the cells infected with CVB3, we identified a cleavage fragment of TRIM56 migrating at ~42 kDa at 24 h and 36 h of p.i. (Fig 8A). Because CVB3 infection induces apoptosis, it is possible that TRIM56 cleavage is the result of apoptosis. Thus, we determined the role of caspases in TRIM56 cleavage in CVB3-infected HEK293T cells. We found that pan-caspase inhibitor Z-VAD-FMK did not block the cleavage of TRIM56 (Fig 8B). The inhibited apoptosis (as shown by reduction of the cleaved caspase-3) had no impact on the appearance of the cleaved TRIM56 fragment (~42 kDa). The slight reduction of 3D in the cells treated with pan-caspase inhibitor is likely due to the inhibitory effect of pan-caspase inhibitor on viral replication [50]. These observations demonstrate that TRIM56 cleavage is induced by CVB3 rather than the activated caspases.

The two enteroviral proteases, 2A^pro^ and 3C^pro^, play critical role in the pathogenesis of the viruses [51]. The cleavage sites of enterovirus 3C^pro^ are typically Gln/Gly, Gln/Ala, or Gln/Ser [41]. Through analyzing the amino acid sequence of TRIM56 (Accession: XM_054359114.1), multiple putative cleavage sites of 3C^pro^ were identified. Therefore, we predicted that TRIM56 is likely cleaved by 3C^pro^ of CVB3. To verify that viral 3C^pro^ cleaves TRIM56, we transfected HEK293T cells with the construct expressing 3C^pro^ and determined the cleavage of TRIM56. We found that expression of 3C^pro^ is sufficient to induce TRIM56 cleavage (Fig 8C). Moreover, the mutated 3C^pro^ (EGFP-3C^mut^ with C147A mutation) with no protease activity failed to cleave TRIM56 (Fig 8C). However, whether or not TRIM56 can also be cleaved by 2A^pro^ of CVB3 needs to be investigated. Furthermore, the *in vitro* interaction between TRIM56 and 3C^pro^ was also determined. As shown in Fig 8D, GST-tagged TRIM56 was expressed in *E*. *coli* and purified by glutathione affinity column. The purified GST-TRIM56 was incubated with the total cellular protein extracted from the cells expressing EGFP-3C. The incubation mixture was subjected to immunoblotting (Fig 8E). We show that cleaved GST-TRIM56 (around 68 kDa) appeared when GST-TRIM56 was incubated with the cell lysate containing EGFP-3C, while cleaved TRIM56 was absent when incubated with the cell lysate containing control vector (pEFGP-C1) (Fig 8E). To further demonstrate that TRIM56 interacts with 3C of CVB3, the subcellular localization of these two molecules was observed by fluorescence microscopy. We show that ectopically expressed 3C and TRIM56 were colocalized in the cytoplasm (Fig 8F), indicating that 3C is the viral protease which interacts with and cleaves TRIM56. Collectively, these data demonstrate that TRIM56 is cleaved by 3C^pro^ of CVB3.

## 3. Discussion

**Fig 9:**
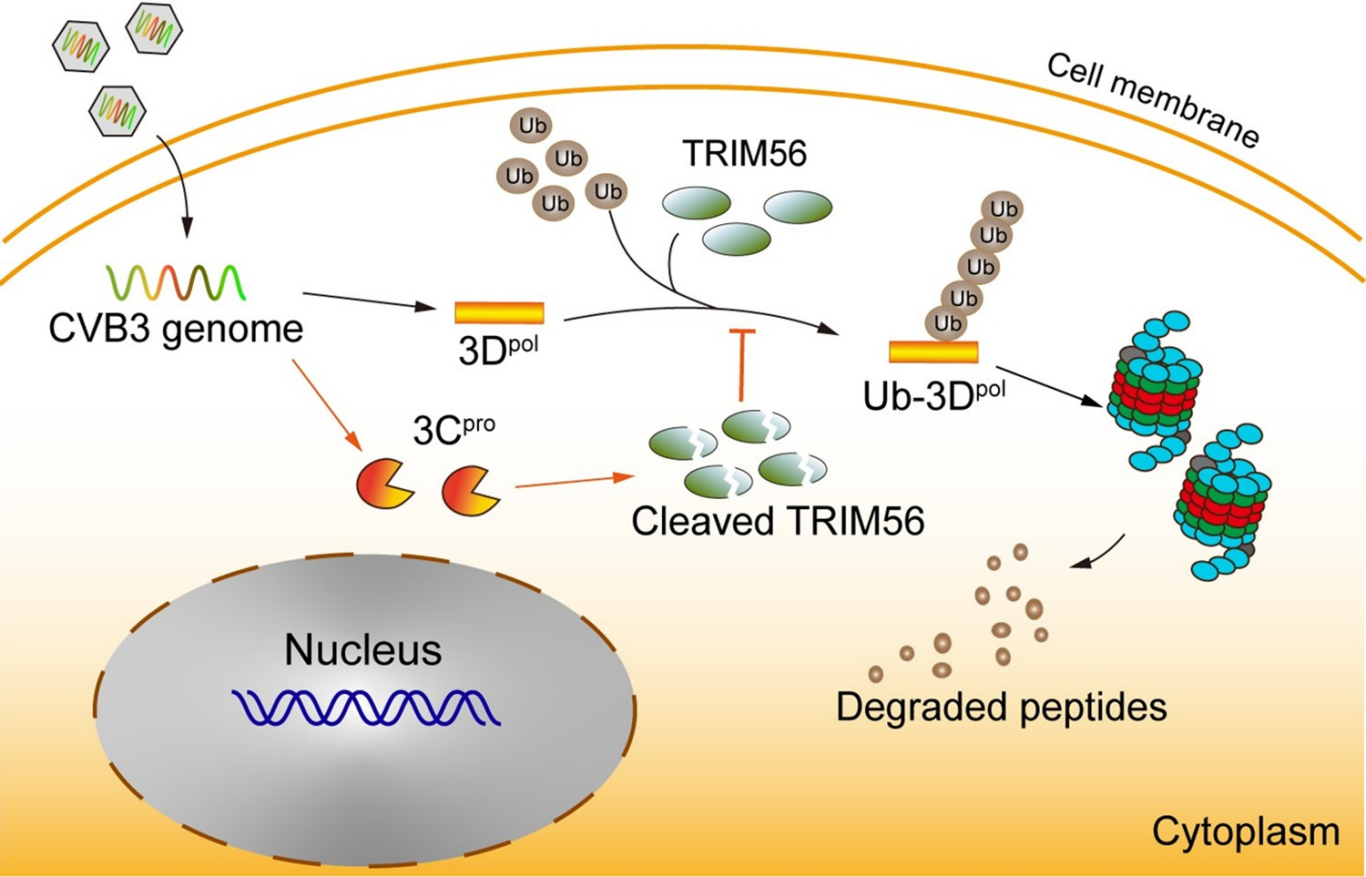
The E3 ligase TRIM56 promotes the ubiquitination and proteasomal degradation of the 3D protein of CVB3. In CVB3-infected cells, TRIM56 interacts with 3D and promotes 3D ubiquitination. Viral protease 3C cleaves TRIM56 to combat TRIM56-medicated 3D degradation.

Ubiquitination is the post-translational modification of proteins which plays essential roles in regulating important cellular functions such as protein stability, innate immunity, and antiviral response [13]. Evidence has shown that viruses can hijack ubiquitination to stabilize viral constituents and facilitate viral replication [52, 53]. In this study, we show that 3D protein, the RdRp of CVB3, is modified at K220 with K48-linked ubiquitin chain. We identified that TRIM56 is the E3 ligase which mediates 3D ubiquitination and proteasomal degradation. We also found that TRIM56 is cleaved by viral 3C^pro^ in CVB3-infected cells, suggesting that viral proteases play critical role in overcoming the cellular defense role of UPS (Fig 9). Moreover, we show that overexpression of TRIM56 significantly inhibited CVB3 replication. These findings demonstrate that TRIM56 is an intrinsic cellular restriction factor against CVB infection, and enhancing viral protein degradation could be a potential strategy to control CVB infection.

To degrade viral proteins through UPS is a cellular defense mechanism by which the host cell combats invading pathogens [20, 54, 55]. On the other hand, viruses such as severe acute respiratory syndrome coronavirus 2 (SARS-CoV-2), Zika virus, and Ebola virus, have adapted cellular ubiquitination mechanism to enhance viral replication or evade innate immunity [12, 14, 36]. The correlation between UPS and CVB infection has long been implicated. Previous studies demonstrated that CVB3 infection upregulated the overall level of cellular ubiquitinated proteins, E1 Ub activating enzyme, E2 Ub-conjugating enzyme UABCH7, and deubiquitinating enzyme UCHL1 [56]. Although the activity of proteasome remained unchanged during CVB3 infection, the use of proteasome inhibitor MG132 attenuated CVB3 replication and myocardial damage [56, 57]. Furthermore, dysregulating UPS with curcumin inhibited CVB3 replication [16]. These findings indicate that UPS is utilized by CVB to enhance viral replication [15]. However, the precise role played by ubiquitination in CVB replication remains largely unknown.

In this study, we began with the measurement of the ubiquitination status of 3D protein in the cells infected with CVB3. We found that CVB3 infection indeed upregulated the overall level of cellular ubiquitination, represented by the accumulation of the ubiquitinated proteins. Importantly, we noted that the 3D protein of CVB3 was likely polyubiquitinated due to the appearance of the 3D protein migrating at high MW (Fig 1B). Enterovirus proteins, including 3D, are processed from high MW viral precursor polyprotein by viral proteases (2A^pro^, 3C^pro^, 3CD^pro^) [58–60]. Therefore, we initially supposed that these 3D-containing proteins with high MW are the processing intermediates of P3 (Fig 1A), which may include 3CD, 3BCD, and 3ABCD (P3). According to the predicated MW of 3A (10 kDa), 3B (2.4 kDa), 3C (20 kDa), and 3D (58 kDa) (GenBank: U57056.1), we estimated that the MW of any of the 3D-containing intermediate precursors (3CD, 3BCD, and 3ABCD) would be less than 100 kDa. Moreover, we found that the banding pattern at 72 kDa to 100 kDa is similar between the blots probed by anti-3D and anti-3C (Fig 1C and 1D), indicating that these proteins contain both 3D and 3C, which are likely 3ABCD, 3BCD, and 3CD, respectively. It should be noted that the separating PAGE gels were different for the blot probed by anti-3D antibody (10% polyacrylamide) (Fig 1C) and that probed by anti-3C antibody (12.5% polyacrylamide) (Fig 1D), which may lead to the slightly different migration rate of these precursors. By comparing the results obtained from the blots using anti-3D and anti-3C, it is obvious that 3D-containing proteins at high MW (>100 kDa) in CVB3-infected cells do not contain the 3C protein of CVB3. Thus, these proteins are not the viral intermediate precursors of P3. Therefore, we proposed that the 3D-containg proteins with high MW in CVB3-infected cells are the polyubiquitinated 3D, since ubiquitination events are abundant in the cell [8]. Denatured IP shows that it is 3D rather than 3CD or 3C that is modified by Ub chain. Ub chain is linked to K220 of the 3D protein in the form of K48 linkage, leading to the proteasomal degradation of 3D. Unlike the previous report that 3D of CVB3 might be modified by monoubiquitination [16], here we show that 3D was polyubiquitinated, since the modified 3D shows MW higher than 180 kDa.

In the process of ubiquitination, Ub is activated by an E1 activating enzyme and transferred to an E2 conjugating enzyme. The E2 either transfers the Ub to a substrate or to an E3 Ub ligase, which then delivers Ub to the substrate [8, 10, 61]. In either case, it is the E3 ligase that determines the specificity of the substrate to be ubiquitinated [62]. However, it is a challenging task to identify the E3 ligase which is responsible for 3D ubiquitination, since there are at least more than 600 E3 ligase genes in human genome and more waiting to be identified [63]. To this end, we analyzed the protein expression profile of the cells infected with CVB3 by mass spectrometry and found that the protein level of TRIM56 was significantly increased while other TRIMs remained largely unchanged. Our further investigations demonstrated that TRIM56 interacts with 3D both *in vitro* and in the cell. The ubiquitination and proteasomal degradation of 3D was upregulated in the cells overexpressing wild type TRIM56, but not the mutated TRIM56, which lacks the E3 ligase activity. When K220, the ubiquitination site of 3D, was mutated, significantly high level of 3D interacting with TRIM56 was identified (Fig 6D; 6H: lane 6 on the top blot). We also show that TRIM56 overexpression significantly down-regulated CVB3 3D protein, while TRIM56 knockdown prolonged the stability of 3D. These data demonstrate that TRIM56 is the E3 ligase which promotes the ubiquitination and proteasomal degradation of the 3D protein of CVB3. On the other hand, we noted that the ubiquitination of 3D was not completely blocked in the cells with TRIM56 knockdown (Fig 7D, lane 3 vs lane 1), suggesting that, in addition to TRIM56, other E3 ligases might also be involved in the ubiquitination of the 3D protein of CVB3. It is also likely that, as an important E3 ligase, TRIM56 may induce various forms of ubiquitination of a variety of cellular proteins, which in turn facilitate or inhibit CVB3 replication. This may explain why CVB3 replication was not significantly altered with TRIM56 knockdown (S1 Fig). The non-significant impact of siTRIM56 on CVB3 replication also suggests that in physiological situation, the ubiquitination of viral 3D protein is unable to restrict CVB3 replication. These data further implicate that there might be countermeasures used by CVB3 to suppress the ubiquitination of viral protein 3D.

The RING domain of TRIM family proteins is usually considered as the E3 ligase domain, while the C-terminal domain of TRIMs functions to recognize and interact with substrate proteins to be ubiquitinated [18]. Unlike the antiviral effect of TRIM56 towards Influenza virus A and B which exclusively depends on its C-terminal tail [33], we show that TRIM56 promotes the K48-linked ubiquitination and proteasomal degradation of 3D of CVB3. Our data indicate that the antiviral effect of TRIM56 relies on its E3 ligase activity, indicating that the molecular integrity of TRIM56 is prerequisite for its anti-CVB3 effect. Therefore, the cleavage of TRIM56 by viral 3C^pro^ is favorable for CVB3 replication. However, we also noted that only a small fraction of TRIM56 was cleaved (Fig 8A and 8B), while the majority of TRIM56 molecules remains intact, suggesting that the E3 ligase function of TRIM56 is largely uninterrupted during CVB3 infection. However, even with the increased expression of TRIM56, viral replication was not suppressed as shown by the increased viral 3D (Fig 8A and 8B), suggesting that there are other mechanisms exploited by CVB3 to restrain the degradative effect of TRIM56 on viral 3D protein. It is highly likely that CVB3 upregulates the expression of deubiquitinating enzymes, which remove the polyubiquitin chain and stabilizes viral 3D protein.

Although the expression of TRIM56 is ubiquitous in various tissues [64], it is also regulated by IFN and viral infection [47, 65]. Our study shows that the abundance of TRIM56 was increased in response to CVB3 infection, while its total protein level remained relatively unchanged during viral replication (from 6 h to 36 h of p.i.) (Fig 4E and 4G). These data were in agreement with the previous report, in which TRIM56 was rapidly increased and remained at relatively high level after poly I:C treatment, while the phosphorylated TRIM56 was found increased in biphasic manner [65]. ERK1/2, which is essential for the proteolytic activity of enterovirus protease 2A [66], is activated in precisely the same manner as that of TRIM56 during CVB3 infection [67]. It remains to be investigated if the rapidly enhanced phosphorylation of TRIM56 occurs during CVB3 infection and if TRIM56 phosphorylation is induced by ERK1/2 activation.

Innate immunity is the first line of cellular defense against viral infection. TRIM56 has been implicated in up-regulating the production of type I IFN through regulating cGAS-STING and TLR3 pathways [68, 69]. It has been demonstrated that CVB3 infection does not elicit pronounced production of type I IFN [49], implicating that upregulated TRIM56 in CVB3-infected cells does not enable cells to activate IFN signaling, which could be at least partly due to the consequence of the 3C^pro^-mediated cleavage of TRIM56, MAVS and TRIF [49]. However, type I IFN signaling is still essential for the control of CVB3 infection, demonstrated by the fact that lack of IFN-β or IFN receptor genes markedly increased the death rate of the mice infected with CVB3 [70, 71]. Moreover, TLR3 deficiency resulted in high virus load and severe myocarditis in the mice infected with CVB3 [72, 73]. These data implicate that the physiological level of proteins in TLR3 pathway is critical for the innate defense system against CVB3 infection. In this study, we show that TRIM56 overexpression markedly suppressed viral progeny production. It is likely that in the context of TRIM56 overexpression, TLR3 mediated IFN signaling, which is upregulated by TRIM56 through interacting with TRIF [69], also contributes to the suppressed viral replication. Thus, further studies are needed to reveal the significance of the up-regulated TRIM56 during CVB3 infection.

According to the previous studies, TRIM56 shows direct antiviral activity towards distinct RNA viruses such as Zika virus, Dengue virus, human Coronavirus, and Influenza virus A and B [32–34]. These studies seem to implicate that the direct antiviral activity of TRIM56 relies on its interaction with specific viral protein. Our results support that the E3 ligase activity of TRIM56 enables its anti-CVB3 effect through interacting with viral 3D protein. Study also showed that TRIM56 is an RNA-binding protein which binds Zika virus RNA through its C-terminal portion, which shares sequence homology to the NHL repeats of TRIM-NHL subfamily proteins [34]. It remains to be investigated whether or not TRIM56 interacts with the genomic RNA of CVB3 and how other members of TRIM-NHL proteins are associated with enterovirus replication.

While ubiquitination plays essential roles in cellular activities and antiviral immunity, it is a dynamic process which is balanced by de-ubiquitination [74, 75]. Although we found that the ubiquitination and degradation of viral 3D of CVB3 was mediated by the E3 ligase TRIM56, we did not investigate whether this process is counterbalanced by specific deubiquitinating enzymes. Considering the fact that CVB3 replication was not suppressed in spite of the increased expression of TRIM56 in the natural course of CVB3 infection, it is likely that the de-ubiquitination of 3D is also elicited by CVB3 infection to maintain the stability of this viral RdRp.

Viruses utilize complex strategies to promote their propagation. Previous study showed the hybrid modification of the 3D protein of EV-A71 with SUMO and K63-linked Ub [76]. Moreover, SUMOylation enhanced EV71 replication by stabilizing 3D protein [76]. Here we show that the 3D polymerase of CVB3 is polyubiquitinated. However, we did not address the question whether or not the 3D protein of CVB3 also harbors other modifications except ubiquitination, which may enhance its stability. Moreover, although we show that TRIM56 was up-regulated in CVB3-infected mouse myocardium, we failed to identify the cleavage fragments of TRIM56, possibly due to that the cleaved fragments are short-lived and do not accumulate in the myocardium.

In summary, we demonstrated that the 3D of CVB3, the viral RdRp, was modified by K48-linked polyubiquitin at K220. The ubiquitination of 3D, which promoted its proteasomal degradation, was mediated by the E3 ligase TRIM56 (Fig 9). Over expression of TRIM56 significantly inhibited viral replication and virus yield. Our study indicates that to modify viral 3D polymerase by ubiquitination is an intrinsic cellular defense strategy against CVB3 infection. A better understanding the interaction between viral proteins and UPS may lead to the development of therapeutic interventions for CVB infection.

## 4. Material and Methods

### 4.1. Ethics statement

The use of laboratory animals in this study was approved by the Ethics Committee of Harbin Medical University. The experiment procedures followed the guidelines for humane animal treatment from the Ethics Committee of Harbin Medical University.

### 4.2. Cell culture

HeLa and HEK293T cells were cultured in Dulbecco’s Modified Eagle Medium (DMEM) (Thermo Fisher, Shanghai, China) supplemented with 10% fetal bovine serum (FBS, Bioindustry, Israel), penicillin (100 U/L), and streptomycin (100 U/L). Cells were incubated in 5% CO_2_ at 37°C and passaged every two days.

### 4.3. Virus

CVB3 Woodruff strain was maintained by the Department of Cell Biology, Harbin Medical University (Harbin, China). The viruses were stored at -80°C, and titrated by 50% tissue culture infectious dose (TCID_50_) or plaque assay. To amplify viruses, HeLa cells were infected with CVB3 for 24 h. Cell cultures were collected and stored at -80°C before the determination of TCID_50_. In this study, the TCID_50_ of CVB3 was 1.1 × 10^7^/mL. HeLa or HEK293T cells were infected with CVB3 at the multiplicity of infection (MOI) of 1.

### 4.4. Mice

The usage of mice was approved and followed by the instructions of Harbin Medical University Ethics Committee on the regulation of laboratory animals. Newborn Balb/c mice were purchased from the Laboratory Animal Center of Harbin Medical University. Mice were housed in individually ventilated caging system and allowed to access to food and water ad libitum in the environment at 24°C with 45% humidity. Mice were infected with CVB3 on day 5 after birth. Control mice were sham-infected with the same amount of DMEM.

### 4.5. Antibodies

Anti-GFP (50430-2-AP), anti-Flag (66008-4-Ig), anti-Caspase3 (19677-1-AP), anti-GAPDH (10494-1-AP) polyclonal antibodies were obtained from Proteintech (Wuhan, China). Anti-TRIM56 (ab154862) and anti-Ub (ab179434) polyclonal antibodies were obtained from Abcam (Cambridge, MA). Anti-DnaK polyclonal antibody was obtained from CUSABIO (Wuhan, China). Anti-3D and Anti-VP1 of CVB3 polyclonal antibodies were prepared in our laboratory.

### 4.6. Plasmid construction

The plasmids expressing EGFP-TRIM56, HA-Ub, HA-Ub-K48, HA-Ub-K48R were purchased from Miaoling (Wuhan, China). The plasmids expressing Flag-tagged 3D of CVB3 (Flag-3D), Flag-3D^1-83^, Flag-3D^84-187^, Flag-3D^188-340^, Flag-3D^341-462^, EGFP-3C, EGFP-3C^mut^ were generated based on pcDNA3.1-EGFP. The lysine residues in Flag-3D^188-340^ were mutated to arginine (R) to generate Flag-3D^188-340^-K220R, Flag-3D^188-340^-K279R, Flag-3D^188-340^-K312R, and Flag-3D^188-340^-K315R. Plasmid pFlag-3D-K220R was constructed to express the full-length of 3D of CVB3 with a mutation at K220 (K to R mutation), based on pFlag-3D. The plasmids expressing the truncated TRIM56, TRIM56-N42 (1–387 aa) and TRIM56-C40 (388–755 aa), were constructed based on pcDNA3.1. The plasmid expressing Flag-tagged 3D of EV-A71, designated as pFlag-3D^A71^, was constructed based on pcDNA3.1-EGFP. The plasmid expressing Myc-tagged 3CD (p3CD-Myc) was constructed based on pcDNA3.1-EGFP.

### 4.7. Transfection

HeLa or HEK293T cells were cultured to 70% confluence in 6-well plates. The transfection mix was prepared with 4 μg plasmid or 25 nmol siRNA (RiboBio, Guangzhou, China) and 3.75 μl Lipofectamine 3000 (Thermo Fisher) dissolved in 1000 μl DMEM. Cells were transfected with transfection mix at 37°C for 4 h and then cultured in fresh medium. Cells were harvested at 24 or 48 h after transfection for further analysis.

### 4.8. Proteasome degradation assay

MG132 (Selleck, Shanghai, China) was dissolved in DMSO to prepare 10 mmol/L stock solution and stored at -20°C. Fresh working solution of MG132 (10 nmol/L) was prepared with DMEM. Six hours before the endpoint of the culture, cells were treated with MG132. Cells were harvested and subjected to Western blotting.

### 4.9. Cycloheximide chase assay

Cycloheximide (CHX) (Abmole, Shanghai, China) was dissolved in DMSO to prepare 20 mg/mL stock solution and stored at -20°C. CHX stock was diluted with DMEM to the working concentration of 20 μg/mL. HEK293T cells were co-transfected with Flag-3D and siTRIM56 for 24 h, followed by the treatment of CHX. Cells were collected at various time points after CHX treatment and subjected to Western blotting.

### 4.10. RNA extraction, reverse transcription and real-time quantitative PCR

Total RNA was extracted by TRIzol (Invitrogen) according to the protocol provided by the manufacturer. RNA was dissolved in nuclease-free water and quantified by Nanodrop 2000 (Thermo Fisher, Waltham, MA). 20 μl reverse transcription system was prepared with 1 μg RNA and 4 μl of 5 × TransScript All-in-One SuperMix (TransGen, Beijing, China). The reverse transcription mix was incubated at 42°C for 15 min and then heated at 85°C for 15 s. Quantitative PCR (qPCR) was carried out on LightCycler 96 (Roche, Basel, Switzerland) with TransStart Top Green qPCR SuperMix (TransGen, Beijing, China). 20 μl PCR reaction system contained 1 μl cDNA, 0.4 μl of each primer (10 μM), and 10 μl of 2 × TransStart Top Green qPCR SuperMix. PCR reaction was carried out for 45 cycles. Each amplification cycle was consisting of denaturation at 94°C for 5 s, annealing at 58°C for 15 s, and extension at 72°C for 1 min. The gene expression level was calculated by 2^-△△Ct^ [77] and normalized by the expression level of GAPDH. Primers used in this study were synthesized by ComateBio (Jilin, China). The sequences of the primers are listed in Table 1.

**Table 1:** Primer sequences.

Primer	Sequence (5′➔3′)
CVB3	Forward: GCACACACCCTCAAACCAGA Reverse: ATGAAACACGGACACCCAAAG
TRIM56	Forward: GCCTGCATA CCTACTGCCAAG Reverse: GCAGCCCATTGACGAAGAAGT
GAPDH	Forward: GGAGCGAGATCCCTCCAAAAT Reverse: GGCTGTTGTCATACTTCTCATGG
TRIM56 (mouse)	Forward: AAGACTCCTCCCCAACTCTG Reverse: GGCAATAGGTATGTAGGCATGG
GAPDH (mouse)	Forward: AGGTCGGTGTGAACGGATTTG Reverse: GGGGTCGTTGATGGCAACA
TRIM9	Forward: GTGTGCGGCTCCTTCTATCG Reverse: GCTGTATAGGCTCATCTTGTCCA
TRIM14	Forward: TGAAGGGGAAATTCACTGAACTC Reverse: AGCCTCTGGACAGGATCGG
TRIM21	Forward: TCAGCAGCACGCTTGACAAT Reverse: GGCCACACTCGATGCTCAC
TRIM22	Forward: CTGTCCTGTGTGTCAGACCAG Reverse: TGTGGGCTCATCTTGACCTCT
TRIM25	Forward: AGCAGCTACAACAAGAATACACG Reverse: GGCTCTGTTCAATCTCCTCCT
TRIM27	Forward: AGCCCATGATGCTCGACTG Reverse: GGGCACGACACGTTAGTCT

### 4.11. Western Blotting

Cells cultured in 6-well plates were washed twice with cold PBS and collected with 100 μl RIPA buffer (Beyotime, Wuhan, China) containing 1% protease inhibitor PMSF (Beyotime). Cells were lysed for 20 min on ice and centrifuged at 12000 rpm at 4°C for 15 min to collect the supernatant. Protein concentration was determined by the BCA protein assay kit (Beyotime). About 20 μg cell lysate was added into 10% polyacrylamide gel (SDS-PAGE) to separate. Immunoblot was carried out to transfer the gel to polyvinylidene difluoride (PVDF) membranes (Millipore, Kenilworth, NJ) under a constant current of 300 mA. PVDF membranes were blocked in skimmed milk for 1 h and incubated with primary antibody for 2 h at room temperature. The membranes were washed with 0.1% Tween-20 in TBST and incubated with secondary antibody for 1 h at room temperature. The blots were treated with ECL (Beyotime) and visualized by Tanon-5200 Chemiluminescent Imaging System (Biotanon, Shanghai, China).

### 4.12. Immunoprecipitation

The linkage between Ub and 3D of CVB3 was identified by immunoprecipitation (IP) performed in denatured conditions. HEK293T cells cultured in 6-well plates were washed twice with cold PBS and then collected with 100 μl cold IP lysis buffer (Proteintech, PK10008) containing 1% protease inhibitor PMSF (Meilunbio, MB12707). Cells were incubated on ice with IP lysis buffer for 30 min and then treated with ultrasound under the power of 180W for 1 min. After the ultrasound treatment, cell lysates were centrifuged at 12000 rpm at 4°C for 15 min to collect the supernatant. Protein concentration was determined by the BCA protein assay kit (Beyotime, P0011). 3 mg of cell lysates were incubated with 4 μg of Flag antibody in 200 μl of incubation buffer at 4°C for 4 h with rotation, followed by the addition of protein A sepharose beads. IP was carried out at 4°C for another 4 h with rotation. The beads were washed five times with IP wash buffer containing 350 mM NaCl. The precipitated proteins were eluted using 80 μl elution buffer. The eluted proteins were separated in 10% SDS-PAGE followed by immunoblotting.

To identify the interaction between TRIM56 and 3D of CVB3, co-IP was performed in undenatured conditions. HEK293T cells cultured in 6-well plates were washed twice with cold PBS and then collected with 100 μl cold IP lysis buffer (Proteintech, PK10008) containing 1% protease inhibitor PMSF (Meilunbio, MB12707) to prepare cell lysates. About 500 μl cell lysate was incubated with 25 μl immunomagnetic beads conjugated with anti-EGFP antibody (Epizyme, Shanghai, China) at room temperature for 1 h with rotation. The precipitated mixture was placed on a magnetic stand for 1 min to remove the supernatant. The magnetic beads were washed with 500 μl rinsing buffer (Epizyme, YJ211) by gently pipetting, followed by 1 min rest on a magnetic stand. Bead wash was repeated 10 times. The precipitated protein complex was eluted with the addition of 100 μl elution buffer (Epizyme, YJ211) and incubation at room temperature for 10 min. The precipitated proteins were analyzed by SDS-PAGE and immunoblotting.

### 4.13. Cell lysate preparation for the analysis of mass spectrometry

HeLa cells were infected with CVB3 at MOI of 1 for 24 h. Culture medium was aspirated and cells were washed with ice-cold PBS. Cell lysates were prepared with IP lysis buffer (Proteintech) supplemented with PMSF. Cell lysates were subjected to the analysis of nanoscale liquid chromatography coupled to tandem mass spectrometry (EASY-nLC 1200, Thermo Fisher).

### 4.14. Fluorescence microscopy

HEK293T cells were grown to subconfluency on coverslips in 24-well plates. Where indicated, cells were transfected with 200 ng of plasmid (pFlag-3D or pFlag-3D-K220R) using Lipofectamine 3000 according to the manufacturer’s protocol for 24 h. Cells were visualized in fluorescence microscopy or visualized after CVB3 infection. For virus infection, 24 h after plasmid transfection, cells were infected with CVB3 at MOI of 1 for 12 h. To visualize cells with fluorescence microscopy, cells were fixed with 4% paraformaldehyde for 20 min at room temperature, followed by permeabilization with PBS containing 0.1% Triton X-100 for 10 min. Cells were then incubated sequentially with primary and secondary antibodies diluted in PBS containing 2% normal goat serum. Viral 3D or 3D-K220R was determined with anti-Flag antibody. Alexa Fluor 488- or Alexa Fluor 594-conjugated goat anti-rabbit and goat anti-mouse (Molecular Probes) were used as secondary antibodies. Nuclei were stained using 4′,6′-diamidino-2-phenylindole (DAPI). Coverslips were mounted with FluorSave (Calbiochem). Images were acquired with Cell Voyager 1000 (Yokogawa, Japan) confocal laser scanning microscope.

### 4.15. Statistical analysis

All the experiments were repeated at least three times. Data were analyzed with GraphPad Prism 9. Student’s *t* test and One Way ANOVA were used to compare the differences between groups. All *P* values were two-tailed. *P* < 0.05 was considered as statistically significant.

## Supporting information

S1 Table Data of mass spectrometry for CVB3-infected cells. HeLa cells were infected or mock-infected with CVB3 at MOI of 1 for 24 h. Culture medium was aspirated and cells were washed with ice-cold PBS. Cell lysates were prepared with IP lysis buffer supplemented with protease inhibitor. Cell lysates were analyzed by nanoscale liquid chromatography coupled to tandem mass spectrometry. Protein abundance which is normalized to GAPDH was provided.
(XLSX)

S1 Fig CVB3 replication is not influenced in the cells with TRIM56 knockdown. HEK293T cells were transfected with siTRIM56 for 24 h, followed by CVB3 infection (MOI of 1) for 12 h. Cells were harvested to determine viral 3D, VP1 (A) and TCID_50_ (B). #: no significant.
(TIF)
