# Supplementary figures and images for: TRIM56 restricts Coxsackievirus B infection by mediating the ubiquitination of viral RNA-dependent RNA polymerase 3D
Source: PLoS Pathog. 2024 Sep 30;20(9):e1012594. doi: 10.1371/journal.ppat.1012594 (PMC11476688; doi:10.1371/journal.ppat.1012594)

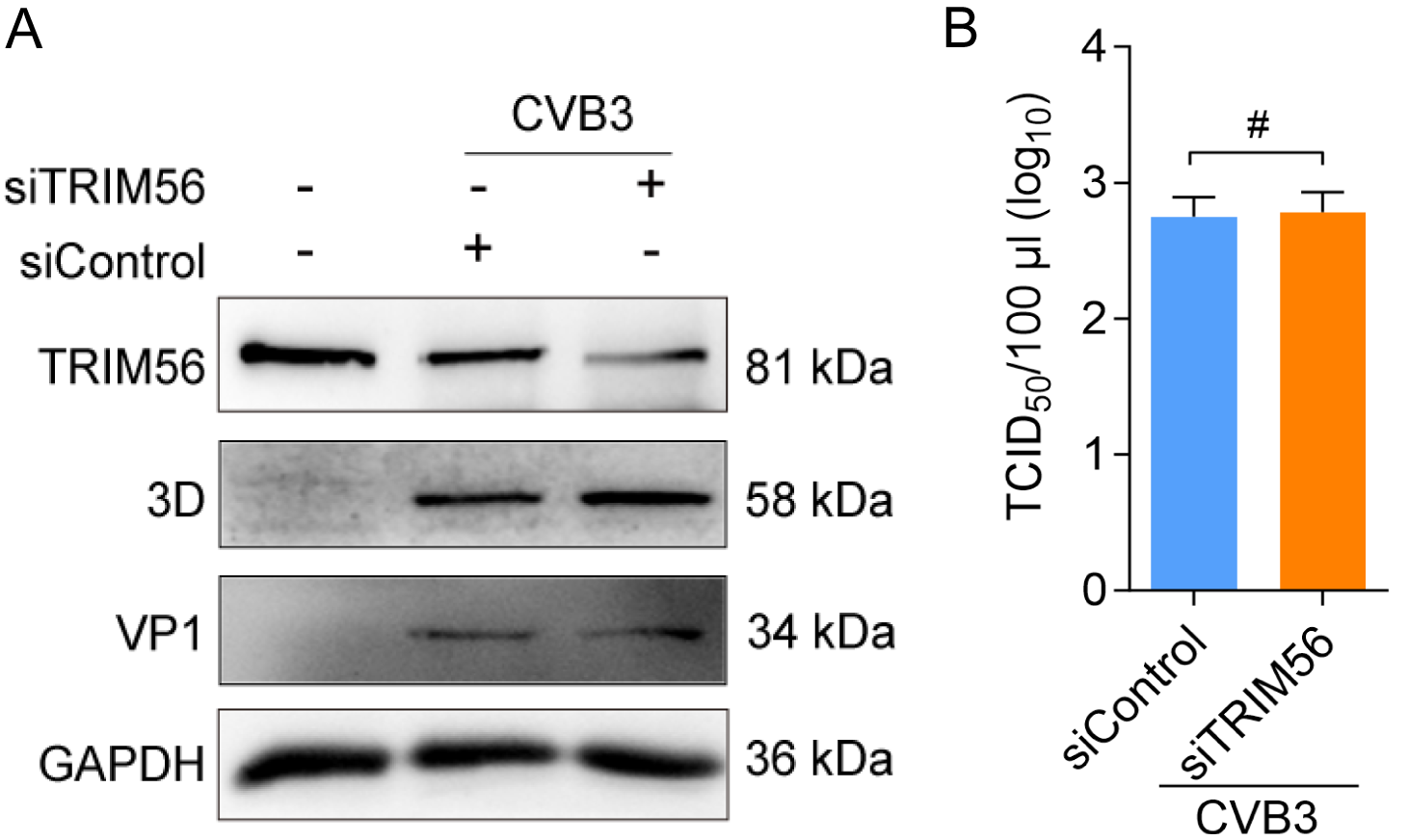

Supplement: S1 Fig — HEK293T cells were transfected with siTRIM56 for 24 h, followed by CVB3 infection (MOI of 1) for 12 h. Cells were harvested to determine viral 3D, VP1 (A) and TCID50 (B). #: no significant. (TIF) [file ppat.1012594.s002.tif]
